# The relationship between hidradenitis suppurativa and irritable bowel syndrome: a cross-sectional study

**DOI:** 10.3906/sag-2107-158

**Published:** 2022-01-09

**Authors:** Abdullah DEMİRBAŞ, Ömer Faruk ELMAS, Hediye EKER, Gözde ULUTAŞ DEMİRBAŞ, Recep DURSUN, Mustafa ATASOY, Ümit TÜRSEN, Torello LOTTİ

**Affiliations:** 1 Department of Dermatology, Kocaeli University, Kocaeli, Turkey; 2 Department of Dermatology, Private Medicana Hospital, İstanbul, Turkey; 3 Department of Dermatology, Selçuk University, Konya, Turkey; 4 Department of Dermatology, Kocaeli State Hospital, Kocaeli, Turkey; 5 Department of Dermatology, Necmettin Erbakan University, Konya, Turkey; 6 Department of Dermatology, Health Science University, Kayseri City Hospital, Kayseri, Turkey; 7 Department of Dermatology, Mersin University, Mersin, Turkey; 8 Department of Dermatology, Guglielmo Marconi University, Rome, Italy; 9 Department of Dermatology, First Medical State University of Moscow I M Sechenev, Moscow, Russia

**Keywords:** Gut, microbiome, dysbiosis, skin, functional bowel disease

## Abstract

**Background/aim:**

Irritable bowel syndrome (IBS) is a functional gastrointestinal disorder in which one experiences abdominal pain, tension, cramping, bloating, and changes in the form and frequency of defecation, without an underlying organic disease. Many skin diseases have been reported to be more common in people with functional bowel disease. To our knowledge, however, no previous study investigated the potential relationship between hidradenitis suppurativa (HS) and IBS. In this study, we aimed to examine the potential association between IBS and HS.

**Materials and methods:**

Patients with HS and healthy subjects were enrolled in this cross-sectional study. All participants were assessed for the presence of IBS. ROME IV criteria were used to identify IBS cases. Hurley staging, modified Sartorius score, and physician’s global assessment score were applied to define clinical severity and staging of HS.

**Results:**

According to the Rome IV diagnostic criteria, 54 (67.50%) of 80 HS patients and 23 (28.75%) of 80 control group were diagnosed with IBS. The frequency of IBS was statistically significantly higher in the patient group than in the control group (P < 0.001). No statistically significant difference was found between the two groups in terms of abnormal stool frequency and family history of IBS (P = 0.28, P = 0.862, respectively). Abnormal stool form, mucus in stool, abdominal distension, feeling of incomplete evacuation were statistically significantly higher in HS patients compared to the controls (P = 0.01, P = 0.02, P < 0.001, P = 0.001, respectively).

**Conclusion:**

Our study revealed that there might be a potential link between HS and IBS.

## 1. Introduction

Hidradenitis suppurativa (HS) is a chronic inflammatory cutaneous disorder of hair follicles delineated by recurrent, painful, nodular lesions and abscesses leading to sinus tracts, fibrosis, and scars. HS prevalence is 1%–4% and often occurs in body areas such as the axilla, inguinal, and/or anogenital areas after puberty [1]. The etiopathogenesis of HS has not been fully elucidated yet. Immunogenetic, hormonal, mechanical stress, smoking, and environmental factors, however, are involved in the formation of the disease. Immunological mechanisms defined in the pathogenesis of HS include the lack of gamma-secretase/Notch signaling pathway, abnormal activation of the T helper 1/17 axis, and dysregulation of the SOX9-Wnt signaling pathway which is located in the active follicular infundibulum and associated with the progression of the hair cycle. Interactions of these immunological paradigms lead to infundibular keratinocyte proliferation and follicular occlusion followed by follicular dilatation, rupture, and inflammation [2–4]. In addition, it has been reported that the number of mast cells in perilesional normal tissue increases in early and chronic HS lesions. Mediators such as histamine and tryptase secreted from these mast cells have been shown to cause hyperkeratinization, which is considered essential for the development of early lesions of HS, by increasing keratinocyte activity through H1 receptor [5]. Another factor in the etiology of HS is diets with a high glycemic index. Diets with a high glycemic index elevate the levels of insulin and insulin-like growth factor-1 (IGF-1) activity, stimulating the mammalian target of rapamycin complex 1 (mTORC1). mTORC1, the pathway associated with food signaling, contributes to follicular duct blockage by inducing overexpression of cytokeratins, hyperproliferation of keratinocytes, and hypercornification of the follicular wall. Besides, mTORC1 activation has been shown to have a fundamental role in the differentiation of Th17 cells, which play a central role in HS pathogenesis [6–8].

Irritable bowel syndrome (IBS) is a functional gastrointestinal disorder distinguished by chronic abdominal pain or discomfort in association with altered bowel habits without specific pathological findings [9]. Its incidence in the general population is about 10% [10].

Although the exact cause of IBS is unknown, genetic, emotional, cognitive, and environmental variables are all thought to have a role. Various pathogenetic mechanisms including mucosal immune system activation, alterations in intestinal motility, visceral hypersensitivity, neuroendocrine disturbance, and brain-intestinal axis abnormalities have been postulated [9,11].

Cutaneous symptoms may accompany IBS, and intestinal dysbiosis, which describes an imbalance of the intestinal microbiota. Intestinal dysbiosis has been linked to the pathogenesis of IBS as well as a number of chronic inflammatory cutaneous conditions such as acne vulgaris, psoriasis, and atopic dermatitis [12–14]. It has been reported that commensal bacteria that form the intestinal microbiota are not properly distributed in patients with HS and result in intestinal dysbiosis [15]. Repair and regeneration signaling pathways including mTORC 1, Notch, Wnt, and TGF-beta have been shown to affect the intestinal microbiota complex by altering cell-cell junctions, secretory cell activity and quantity, and intestinal barrier permeability [16]. Along with the dysregulation of these signaling pathways, increased number of mast cells, inflammation, and diet with high glycemic index play roles in both IBS and HS pathogenesis [2–9,11,17]. We postulated that there could be a link between HS and IBS based on these common pathogenetic pathways and the present study aims to investigate this potential relationship.

## 2. Materials and methods

### 2.1. Subjects

This study included patients with HS and age-gender matched healthy subjects who were admitted to a public hospital’s outpatient dermatology clinic between June 2020 and September 2020.

### 2.2. Inclusion and exclusion criteria

Patients and controls over the age of 18, who did not receive probiotic-prebiotic food supplements in the last 3 months, did not apply a special diet, did not have an accompanying infection, cancer, cutaneous and systemic disorders were enrolled in the study. Patients and controls who were treated with any topical or systemic agents during the previous three months and healthy volunteers with a family history of HS were excluded. Furthermore, patients with HS who were diagnosed with inflammatory bowel disease via colonoscopy were excluded from the study.

### 2.3. Diagnosis and staging

The diagnosis of HS was based on previously defined clinical criteria, including three key elements [18,19].

1. Typical lesions: The presence of inflamed or noninflamed nodules, sinus tracts, abscesses, and scarring
2. Characteristic distribution: Involvement of axilla, genitofemoral region, perineum, gluteal region, and inframammary area of women
3. Recurrence: Recurrent painful or suppurating lesions more than twice in a period of six months

Demographic features of all participants were recorded. For each patient, the duration, severity, and family history of HS were reviewed. The severity and staging of HS were measured applying the Hurley staging [18], modified Sartorius score [20], and physician’s global assessment score [21].

All the participants were evaluated for the presence of IBS. ROME IV diagnostic criteria were used for the diagnosis [9]. The findings supporting the IBS diagnosis such as the frequency of evacuation, frequency of abnormal stool, forms of abnormal stool, mucous in the stool, abdominal distention, and sense of incomplete evacuation were also reviewed for both groups. In addition, the participants were asked to identify the form and consistency of their stools using the Bristol stool scale [22]. Patients were also asked if they had an increase in HS symptoms during IBS episodes. Participants who reported “alarm symptoms” were excluded from the study and sent to a gastroenterologist [23].

### 2.4. Statistical analysis

The statistical analyses were performed using IBM SPSS Statistics version 23 software (SPSS Inc., Chicago, IL, USA). Descriptive statistical parameters included number, percentage, mean, standard deviation, median, and IQR. The Kolmogorov-Smirnov test was used to test whether the data were distributed normally. Relationships between variables were evaluated using chi-square test and Mann-Whitney U test. Results were evaluated at a 95% confidence interval; and a p < 0.05 value was recognized as the statistical significance level.

### 2.5. Ethics approval

All the procedures followed the Helsinki declaration and the study was approved by local Institutional Review Board (decision date and number: 2020-13/103). Written informed consent was obtained from all the participants.

## 3. Results

A total of 80 patients with HS (45 females, 35 males) and 80 (44 females, 36 males) age and gender-matched healthy subjects were enrolled in the study (p = 0.874). The mean ages of the patients and controls were 34.55 ± 4.67 and 34.53 ± 6.87, respectively (p = 0.928). The mean disease duration was 11.01 ± 5.23 years. The mean HS modified Sartorius score is 18.63 ± 14.53. According to Hurley staging, 43 (53.75%) patients were in stage 1, 29 (36.25%) were in stage 2, and 8 (10%) were in stage 3. According to the physician’s global assessment, 43 (53.75%) patients had mild disease, 29 (36.25%) had moderate disease, 4 (5%) had severe disease, and 4 (5%) had very severe disease. HS was accompanied by severe acne in 34 (42.50%) patients and pilonidal sinus in 13 (16.25%) patients. Twenty-two (27.50%) patients had a family history of HS. The demographic and clinical features of both groups are demonstrated in table 1.

Based on the Rome IV criteria, 54 patients (67.5%) and 23 healthy subjects (28.75%) received a diagnosis of IBS. Compared to controls, patients with HS showed a statistically significantly higher frequency of IBS (P < 0.001). The frequency of abnormal stool and family history of IBS showed no statistically significant difference between the two groups (P = 0.28, P = 0.862, respectively). The frequencies of abnormal stool form, mucus in stool, abdominal distension, and sense of incomplete evacuation were statistically significantly higher in HS patients compared to the control group (P = 0.01, P = 0.02, P < 0.001, P = 0.001, respectively). HS lesions were furthermore evaluated in terms of exacerbation during IBS episodes. Forty-two (77.77%) of the patients did not report any exacerbation while 12 (22.22%) described aggravation of the symptoms related to HS during IBS episodes (Table 2). Bristol stool form scale showed no statistically significant difference between the two groups (P = 0.34) (Table 3).

The relationship between modified Sartorius score, age, duration of disease, Bristol stool form scale, abnormal stool frequency, abnormal stool form, mucus in stool, abdominal distension, and feeling of incomplete evacuation was also analyzed. A significant correlation between HS severity and abdominal distension and the Bristol stool form scale was found (r = 0.073, r = 0.028, respectively). In addition, a significant correlation was observed between disease duration and the Bristol stool form scale (r = 0.076) (Table 4).

## 4. Discussion

Our study demonstrated that IBS is more common in patients with HS than those without. Moreover, the findings supporting the diagnosis of IBS such as abnormal stool form, mucus in stool, abdominal distension, and feeling of incomplete evacuation were more prevalent in patients with HS.

HS is not considered a skin-limited disease but a disorder with immune dysregulation and systemic inflammation. Two different models have been suggested for the pathogenesis of HS. The key trigger in the first pathogenic model is infundibular follicular occlusion while the second one has mainly focused on autoinflammation. The infundibular follicular occlusion model proposes that deficiencies in gamma-secretase activity and Notch signaling pathway cause infundibular keratinocyte proliferation and follicular occlusion, resulting in follicular dilatation, rupture, and ultimately inflammation. The autoinflammatory hypothesis places inflammation as the fundamental driver of pathogenesis. According to this hypothesis, dermal inflammation caused by diverse contributing factors triggers secondary follicular occlusion, resulting in proliferative gelatinous mass and tunnel formation [2–4].

IBS is a functional gastrointestinal disorder and the diagnosis is mainly based on clinical findings. No sensitive structural or biochemical markers have been identified yet for the diagnosis of IBS [9,24]. In the present study, we used ROME-IV criteria along with supporting clinical findings for the diagnosis of IBS. Although the pathophysiology of IBS is not fully known, many factors are thought to be involved, including intestinal dysbiosis, small intestinal bacterial overgrowth, visceral hypersensitivity, intestinal mucosal immune activation, dietary intolerance, increased intestinal permeability, disruption of the brain-intestinal axis, and psychosocial disturbances [9,11].

Recent studies have revealed that intestinal microbiota has a significant impact on human health. It has been suggested that disruption of the delicate balance between intestinal homeostasis and microbiota may contribute to the development of serious pathologies. The intestinal microbiome regulates the host immune system by protecting the host against exogenous pathogens through activating immunoprotective responses. Therefore, intestinal dysbiosis which describes microbial imbalance, may lead to the development of autoimmune and inflammatory diseases even in various organs distant from gut, including skin. This has led to the recognition of the gut-skin axis and brought new perspectives for the management of different inflammatory diseases [14].

The association between HS and inflammatory bowel diseases has been well established for a long period of time. It has been reported that consultation with gastroenterologists is required when patients with HS present with recurrent abdominal pain, chronic diarrhea, bloody stools, and weight loss [25]. Patients with alarming findings such as chronic diarrhoea, bloody stools, and weight loss were excluded from our study [23]. Additionally, colonoscopy was performed on all patients with HS prior to the study to rule out inflammatory bowel disease.

IBS has been demonstrated to be associated with high frequency of various conditions including asthma, food, pollen and animal allergies, rheumatoid arthritis, as well as some cutaneous disorders [26–28]. Ekiz et al., [27] reported that, compared to controls, patients with chronic itching of unknown origin were more likely to have IBS. Sholam et al., [28] found a similar relationship between IBS and chronic urticaria. Chronic inflammatory diseases such as psoriasis, acne vulgaris, atopic dermatitis, and rosacea have also been demonstrated to be associated with a higher prevalence of IBS, compared to healthy subjects [26,29–31]. The relationship between cutaneous diseases and IBS is attributed to intestinal dysbiosis, which is thought to play a role both in IBS and chronic inflammatory disorders [12–14]. Supporting the relationship between IBS and HS, commensal bacteria of the intestinal microbiota have been reported not distributed properly in patients with HS [15]. Furthermore, increased mast cell count, inflammation, a high glycemic index diet, dysregulation of signaling pathways like mTORC 1, Notch, Wnt, and TGF-beta, and increased serum proinflammatory cytokines like IL-1, IL-6, and TNF-a all play a role in the etiopathogenesis of both HS and IBS [2–9,11,16,17].

We believe that the presence of shared pathogenetic characteristics in both HS and IBS explains why the HS study group had a higher prevalence of IBS in our study.

Our study has three main limitations that need to be addressed. First, the relatively small sample size may limit the generalization of the results. Second, because both groups have a history of smoking and stress, this may contribute to the high incidence of IBS, and the inability to exclude these factors is one of the study’s limitations. Third, it was not possible to determine the actual incidence of IBS in patients with HS due to the cross-sectional study design of the study. Hence, prospective studies with a larger sample size are warranted to deeply understand the relationship between IBS and HS.

In conclusion, our study demonstrated that there may be a relationship between HS and IBS, possibly due to a similar pathogenetic mechanism. We encourage more studies particularly focusing on the common pathogenesis of HS and IBS, which may open a new horizon for the treatment of both entities. To the best of our knowledge, our study is the first to investigate the relationship between HS and IBS.

## Figures and Tables

**Table 1:** Demographic and clinical features of patients group and controls group.

	Patients (N = 80)	Controls (N = 80)	P-value
**Gender, N (%)**			
**Male**	35 (43.75%)	36 (45%)	0.874
**Female**	45 (56.25%)	44 (55%)	
**Age, mean ± Sd**	34.55 ± 4.67	34.53 ± 6.87	0.928
**BMI, mean ± Sd**	27.63 ± 1.46	27.60 ± 1.55	0.924
**Waist circumference, mean ± Sd**	93.89 ± 15.12	93.20 ± 14.69	0.771
**Smoking**, **N (%)**	55 (68.75%)	36 (45%)	**0.002**
**Disease duration (years), mean ± Sd**	11.01 ± 5.23	-	-
**Modified Sartorius score, mean ± Sd**	18.63 ± 14.53	-	-
**Hurley Staging, N (%)**			
**Stage 1**	43 (53.75%)	-	-
**Stage 2**	29 (36.25%)		
**Stage 3**	8 (10%)		
**Physician’s global assessment score, N(%)**			
**Mild**	43 (53.75%)	-	-
**Moderate**	29 (36.25%)		
**Severe**	4 (5%)		
**Very severe**	4 (5%)		
**Additional diseases, N (%)**			
**Severe acne**	34 (42.5%)	-	-
**Pilonidal sinus**	13 (16.25%)		
**None**	33 (41.25%)		
**Hidradenitis suppurativa family history, N (%)**	22 (27.50%)	-	-

BMI Body Mass Index, Sd Standard deviation, P < 0.05 is defined as statistically significant and shown in bold.

**Table 2:** Evaluation of frequency of irritable bowel syndrome and the findings supporting the diagnosis of irritable bowel syndrome in patients and controls.

	Patients (n = 80) N (%)	Controls (n = 80) N (%)	P-value
Frequency of IBS			
(−)	26 (32.50%)	57 (71.25%)	**<0.001**
(+)	54 (67.50%)	23 (28.75%)	
Abnormal stool frequency			
(−)	63 (78.75%)	66 (82.50%)	0.28
(+)	17 (21.25%)	14 (17.50%)	
Abnormal stool forms			
(−)	56 (70.00%)	69 (86.25%)	**0.01**
(+)	24 (30.00%)	11 (13.75%)	
Mucus in stool			
(−)	58 (72.50%)	73 (91.25%)	**0.02**
(+)	22 (27.50%)	7 (8.75%)	
Abdominal distension			
(−)	29 (36.25%)	59 (73.75%)	**< 0.001**
(+)	51 (63.75%)	21 (26.25%)	
The feeling of incomplete evacuation			
(−)	39 (48.75%)	60 (75.00%)	**0.001**
(+)	41 (51.25%)	20 (25.00%)	
Family history of IBS			
(−)	67 (83.75%)	69 (86.25%)	0.862
(+)	13 (16.25%)	11 (13.75%)	
Association of HS and IBS episodes			
(−)	42 (77.77%)	-	-
(+)	12 (22.22%)		

IBS irritable bowel syndrome, HS Hidradenitis suppurativa, P < 0.05 is defined as statistically significant and shown in bold.

**Table 3:** Distribution of ‘Bristol Stool Form’ assessment by groups.

Bristol stool form scale	Patients (n = 80) N (%)	Controls (n = 80) N (%)	P-value
**1**	7 (8.75%)	2 (2.50%)	0.34
**2**	9 (11.25%)	4 (5.00%)	
**3**	26 (32.50%)	26 (32.50%)	
**4**	34 (42.50%)	40 (50.00%)	
**5**	1 (1.25%)	3 (3.75%)	
**6**	2 (2.50%)	3 (3.75%)	
**7**	1 (1.25%)	2 (2.50%)	

**Table 4:** Correlation analysis between age, modified Sartorius score, disease duration, abnormal stool frequency, mucus passage, abdominal distension, incomplete defecation feeling, Bristol stool form scale in the patient group.

	**Age**	**Modified Sartorius score**	**Disease duration**	**Abnormal stool frequency**	**Abnormal stool form**	**Mucus in stool**	**Abdominal distention**	**The feeling of incomplete evacuation**	**Bristol stool form scale**
**Age**									
***r***	1.000	0.492	0.790	−0.200	−0.076	−0.191	−0.220	0.290	0.127
***p***	0.000	0.000004	0.000	0.075	0.500	0.090	0.844	0.790	0.260
**Modified Sartorius Score**									
***r***	0.492	1.000	0.538	−0.121	−0.095	−0.014	0.073	−0.134	0.028
***p***	0.000004	0.000	0.000	0.287	0.402	0.905	0.520	0.238	0.807
**Disease duration**									
***R***	0.760	0.538	1.000	−0.178	−0.143	−0.061	−0.168	−0.260	0.176
***p***	0.000	0.000	0.000	0.114	0.205	0.593	0.136	0.816	0.119
**Abnormal stool frequency**									
***r***	−0.200	−0.121	−0.178	1.000	0.488	0.158	0.279	0.252	−0.146
***p***	0.075	0.287	0.114	0.000	0.000004	0.161	0.012	0.022	0.196
**Abnormal stool form**									
***r***	−0.076	−0.095	−0.143	0.488	1.000	0.024	0.437	0.256	−0.190
***p***	0.500	0.402	0.205	0.000004	0.000	0.830	0.000051	0.022	0.091
**Mucus in stool**									
***r***	−0.191	−0.014	−0.061	0.158	0.024	1.000	0.115	−0.015	0.14
***p***	0.090	0.905	0.593	0.161	0.830	0.000	0.310	0.892	0.214
**Abdominal distention**									
***R***	−0.022	−0.073	−0.168	0.279	0.437	0.115	1.000	0.409	−0.015
***P***	0.844	0.520	0.136	0.012	0.000051	0.310	0.000	0.000165	0.893
**The feeling of incomplete evacuation**									
***R***	0.029	−0.134	−0.026	0.252	0.256	−0.015	0.409	1.000	0.005
***p***	0.799	0.238	0.816	0.024	0.022	0.892	0.000165	0.000	0.967
**Bristol stool form scale**									
***r***	0.127	0.028	0.176	−0.146	−0.190	0.140	−0.015	0.005	1.000
***p***	0.260	0.807	0.119	0.196	0.091	0.214	0.893	0.967	0.000
